# Comparing Effects of Reward Anticipation on Working Memory in Younger and Older Adults

**DOI:** 10.3389/fpsyg.2018.02318

**Published:** 2018-11-28

**Authors:** Franka Thurm, Nicolas Zink, Shu-Chen Li

**Affiliations:** ^1^Chair of Lifespan Developmental Neuroscience, Faculty of Psychology, Technische Universität Dresden, Dresden, Germany; ^2^Chair of Cognitive Neurophysiology, Faculty of Medicine Carl Gustav Carus, Technische Universität Dresden, Dresden, Germany

**Keywords:** working memory, reward modulation, aging, cognitive processing fluctuations, EZ-diffusion model

## Abstract

Goal-directed behavior requires sufficient resource allocation of cognitive control processes, such as the ability to prioritize relevant over less relevant information in working memory. Findings from neural recordings in animals and human multimodal imaging studies suggest that reward incentive mechanisms could facilitate the encoding and updating of context representations, which can have beneficial effects on working memory performance in young adults. In order to investigate whether these performance enhancing effects of reward on working memory processes are still preserved in old age, the current study aimed to investigate whether aging alters the effects of reward anticipation on the encoding and updating mechanisms in working memory processing. Therefore, a reward modulated verbal *n*-back task with age-adjusted memory load manipulation was developed to investigate reward modulation of working memory in younger (age 20–27) and older (age 65–78) adults. Our results suggest that the mechanism of reward anticipation in enhancing the encoding and updating of stimulus representations in working memory is still preserved in old age. EZ-diffusion modeling showed age distinct patterns of reward modulation of model parameters that correspond to different processes of memory-dependent decision making. Whereas processes of memory evidence accumulation and sensorimotor speed benefited from reward modulation, responses did not become more cautious with incentive motivation for older adults as it was observed in younger adults. Furthermore, individual differences in reward-related enhancement of decision speed correlated with cognitive processing fluctuation and memory storage capacity in younger adults, but no such relations were observed in older adults. These findings indicate that although beneficial effects of reward modulation on working memory can still be observed in old age, not all performance aspects are facilitated. Whereas reward facilitation of content representations in working memory seems to be relatively preserved, aging seems to affect the updating of reward contexts. Future research is needed to elucidate potential mechanisms for motivational regulation of the plasticity of working memory in old age.

## Introduction

Understanding the interactions between cognitive control and motivation has attracted much research attention over the past decade (for a recent review see an edited volume by Braver, 2015). Goal-directed behavior requires efficient resource allocation of cognitive control processes, such as the ability to select or prioritize relevant over less relevant information in working memory. Working memory entails simultaneous maintenance and processing (manipulation) of task-relevant information in the present moment (Baddeley, 2012). Empirical evidence and theories of working memory suggest that basic processes during information encoding and retrieval that involve attention, memory updating, and inhibition support working memory performance. The role of selective attention is particularly elaborated in theories that consider multiple embedded components of working memory (Oberauer, 2002; Oberauer and Hein, 2012; cf. Cowan, 2001), with attention regulating the access to task-relevant information in activated long-term memory with a broad or narrow range of focus.

In terms of the neurocognitive architecture of working memory, research in the past two decades has revealed that the maintenance and processing of information in working memory involve a broad brain network with interactions between frontal, posterior, and sub-cortical regions (cf. Eriksson et al., 2015 for review). In the prefrontal-parietal network, regions in the parietal cortex seem to be involved in selective and sustained attention mechanisms for maintaining information in working memory, which are associated with working memory capacity (Awh et al., 2006). In contrast, the retrieval of task-relevant information which also requires attention seems to depend on prefrontal regions (e.g., Postle, 2006; Darki and Klingberg, 2014). Furthermore, inputs to the prefrontal cortex from striatal regions which are modulated by the neurotransmitter dopamine seem to be associated with the filtering of irrelevant and the selection of relevant information during working memory encoding and retrieval (McNab and Klingberg, 2008). Since the striatal dopamine system is also known to play important roles in regulating reward processing (see Schultz, 2013, 2015 for reviews), incentive motivation may thus influence the selective encoding, updating and retrieval of relevant information. Furthermore, given age-related decline in both working memory and dopamine modulation (see next section for details), a better understanding of adult age differences in the effects of incentive motivation on working memory is of specific interest. In the following, we first present empirical evidence for aging-related working memory decline, followed by a brief review of current findings on reward modulation of cognition, before presenting the aims and hypotheses of the current study.

### Working Memory in Old Age

It is well established that working memory undergoes significant aging-related decline (e.g., Park et al., 2002; Hertzog et al., 2003; Wager and Smith, 2003; Emery et al., 2008; Gajewski and Falkenstein, 2014). Older age is associated with reduced working memory capacity in general (cf. Bopp and Verhaeghen, 2018 for a recent meta-analysis). More specifically, it has been repeatedly demonstrated that the aging-related deficit in working memory is larger for item manipulation than for item maintenance (e.g., Li et al., 2008) and that maintenance of items in working memory is relatively spared in old age unless the amount of distraction or working memory load are increased (e.g., Gazzaley et al., 2007). One commonly used paradigm to investigate working memory processes (cf. Owen et al., 2005 for meta-analysis) and age-related differences in working memory performance (Kirchner, 1958; cf. Bopp and Verhaeghen, 2018) is the *n*-back task. In line with the theoretical framework of multiple-embedded components of working memory (Cowan, 2001; Oberauer, 2002; Oberauer and Hein, 2012), previous research has shown that the necessity of switching attentional focus with increasing *n*-back load reduces the *n*-back performance level more strongly in older compared to younger adults, but with different effects on the accuracy and speed of working memory: With increasing *n*-back load age differences in accuracy increase, whereas age differences in response time seem to remain relatively stable (e.g., Dobbs and Rule, 1989; Verhaeghen and Basak, 2005; Vaughan et al., 2008). Further empirical evidence indicates that working memory deficits in older age are correlated with deficient suppression of distracting and irrelevant information (Gazzaley et al., 2005). Here also selective attention may play a role, as it may help to shield memory content from interference (Oberauer and Lin, 2017; cf. Gazzaley and Nobre, 2012 for review).

Nonetheless, even in old age substantial plasticity of working memory performance is still preserved, allowing older adults to benefit from cognitive training (Li et al., 2008; cf. Karbach and Verhaeghen, 2014 for a meta-analysis) and non-invasive brain stimulation (cf. Passow et al., 2017 for review). For example, training-induced improvements in working memory have been associated with alterations in prefrontal-parietal and prefrontal-striatal network activations and with changes in dopamine receptor density (cf. Klingberg, 2010 for review). However, even after controlling for individual differences in baseline working memory capacity, older adults benefit less from training, which reveals that the plasticity of working memory is more limited in older compared to younger adults (Rhodes and Katz, 2017).

The accumulated evidence from neurocognitive studies supports the idea that age-related decline in working memory arises, in part, from a decrease in neural efficiency that is accompanied by compensating mechanisms (i.e., the recruitment of more neural units) in order to supply the required processing demands for the given tasks. For instance, decreasing neural efficiency in working memory networks has been associated with delays in event-related potential (ERP) indices of working memory processes (Pelosi and Blumhardt, 1999; Missonnier et al., 2011; Gajewski and Falkenstein, 2014) as well as with decreased electrophysiological brain oscillatory activity in the theta and alpha frequency bands (Missonnier et al., 2011; Gajewski and Falkenstein, 2014). Compensatory mechanisms have been observed in terms of over-recruitment of functional activity within the working memory network already at lower levels of working memory load (Emery et al., 2008; Nagel et al., 2011). At the neurochemical level, aging-related deficits in working memory are associated with deficient dopamine modulation of the frontal-striatal circuitry. It is well known that various aspects of the dopamine system progressively decline during the course of aging (for reviews see Bäckman and Farde, 2005; Li and Rieckmann, 2014). Specifically, negative age differences in the binding potentials of striatal presynaptic dopamine transporters (Erixon-Lindroth et al., 2005), frontal (Kaasinen et al., 2000) and striatal (Kaasinen and Rinne, 2002) postsynaptic dopamine D2 receptors are well established. At the computational level, a theory of aging neuronal gain control (Li et al., 2001) elucidates a link between deficient dopamine modulation in old age and increased random processing fluctuations that lead to reduced representational distinctiveness (Li et al., 2001, 2006) with negative consequences on working memory (Li and Sikström, 2002). At the empirical level, evidence from receptor imaging studies shows that striatal dopamine synthesis capacity (Landau et al., 2008) as well as striatal and frontal dopamine D1 receptor binding (Bäckman et al., 2011) contribute to individual and aging-related differences in working memory. Furthermore, aging-related loss in striatal dopamine D1 receptors partly contributes to attenuated fronto-parietal functional connectivity during working memory (Rieckmann et al., 2011).

### Reward Modulation of Cognition

Much recent research effort has been devoted toward understanding the interactions between motivation and cognition (Braver, 2015). Flexible behavior requires efficient allocation of cognitive control processes, such as the ability to prioritize relevant over less relevant information in working memory. Incentive motivation is one factor that could guide such prioritizations. More specifically, the anticipation of a reward during a variety of cognitive tasks can up-regulate the allocation of limited cognitive resources, such as selective attention, for task-relevant processes (Krawczyk et al., 2007; Locke and Braver, 2008; Rowe et al., 2008; Kennerley and Wallis, 2009; Beck et al., 2010; Braver et al., 2014).

At the neuronal level, the dopaminergic mesocortical pathway has been suggested to play an important role in regulating the interactions between motivation and cognition in general (cf. Berridge and Arnsten, 2015; Cools, 2011 for reviews) as well as working memory in specific (e.g., Badre, 2012; D’Ardenne et al., 2012). Thus far, striatal dopamine modulation of motivation-relevant processes (e.g., reward anticipation as well as signaling anticipation-outcome discrepancy, reward magnitude and probability) is well established (cf. Schultz, 2013, 2015 for reviews). Of particular interest, evidence from animal research shows that dopamine neurons also respond to unrewarded stimuli if these were presented in previously rewarded contexts and are, hence, associated with potential future reward. This suggests that, at a more general level, midbrain dopamine is also important for establishing higher-order generalizations of reward contexts (Kobayashi and Schultz, 2014). Such a mechanism might lead to a facilitation of responses to stimuli presented in contexts where rewards are anticipated, albeit the stimuli themselves have previously not been rewarded. In a similar manner, it may be expected that reward cues presented prior to the to-be-remembered stimuli can facilitate the encoding and updating of reward-cued stimuli by activating the reward context.

At the neurocognitive level, it has been shown that in younger adults reward cueing increases prefrontal activity as well as amplifies the enhancement and suppression of visual cortex activity during a working memory task with scenes and faces as stimuli (Krawczyk et al., 2007). Reward manipulation was also found to be associated with a greater electrophysiological parietal P3 component, indicating increased working memory involvement during task performance, and facilitated response time in younger adults (Capa et al., 2013). At the behavioral level, past research in younger adults has shown that reward incentives yield benefits in different aspects of working memory performance, such as accuracy, response time, and response time variability (Epstein et al., 2011; for reviews see Schultz, 2013, 2015). Reward anticipation has also been shown to significantly reduce negative effects of interference and decay during working memory encoding, presumably through motivational modulation of selective attention and sensory saliency (Klink et al., 2017). Furthermore, beneficial effects of incentive motivation on working memory have been found to be comparable for secondary (monetary) and primary (liquid) rewards (Beck et al., 2010).

Thus, in young-adult samples there is clear evidence indicating that incentive motivation can facilitate working memory. However, studies investigating the effects of aging on reward modulation of cognition so far only focused on visual attentional mechanisms (e.g., Spaniol et al., 2011; Störmer et al., 2014) or episodic memory (e.g., Castel et al., 2011, 2013; Chowdhury et al., 2012; Spaniol et al., 2013). For instance, studies using a value-directed remembering paradigm (i.e., to-be-remembered memory items are cued with specific values indicating the points that could be earned if items are correctly recalled during the retrieval phase) revealed that both younger and older adults allocated more study time for high-value items (Castel et al., 2011, 2013). However, the value-based encoding selectivity was lower in older-old adults (80 to 96 years of age) than in other age groups across the adult lifespan (18 to 79 years of age). Regarding visual attention, a study using the visual search paradigm showed that stimulus features (e.g., color) that signaled a higher reward value speeded up visual search in both younger and older adults. Nevertheless, this benefit saturated in later trials, during which older adults’ visual search time benefited less from feature-based reward cueing relative to the young adults. Furthermore, feature-based reward cueing reduced processing fluctuation (e.g., assessed with the coefficient of intraindividual response time variability) in younger adults but not in older adults (Störmer et al., 2014).

### Study Aims and Hypotheses

The current study extends prior research on motivational regulation of cognition. Specifically, since the question of whether the performance enhancing effect of reward on working memory is preserved in old age is still open, we aimed to investigate whether aging may alter effects of reward anticipation on working memory performance in the *n*-back task, especially when *n*-back load is high. To not confound the effects of reward anticipation with negative age differences in baseline *n*-back performance level, we manipulated working memory load in an age-adjusted manner (cf. Emery et al., 2008) using a reward incentivized *n*-back task that we designed. As evidence from animal studies shows that the faster phasic dopamine signaling plays a crucial role in focused reward-related behavioral adaptations (cf. Grace, 2000; Schultz, 2002, 2013; Schultz et al., 2017 for reviews), the reward manipulation was applied continuously at the trial level, thereby inducing a stronger involvement of phasic rather than tonic reward responses (see “Materials and Methods” section for details).

In light of previous empirical findings reviewed above, we expected reward benefits on working memory in both adult age groups. However, given (i) dopamine’s key role in incentive motivational mechanisms and (ii) aging-related decline in different aspects of the dopamine system, we also expected the beneficial effect of reward anticipation on *n*-back performance to be smaller in older compared to younger adults. Since striatal inputs to the prefrontal cortex that are modulated by dopamine have been associated with the selective filtering of irrelevant from relevant information (McNab and Klingberg, 2008) as well as the updating of context representation (D’Ardenne et al., 2012) during working memory encoding and retrieval, we assumed that age differences in the benefits of reward anticipation on working memory would reflect differences in incentive modulation of these basic processes of working memory.

In order to obtain a more fine-grained view on the differential effects of reward anticipation on *n*-back task performance in younger and older adults, we further applied the EZ-diffusion model (cf. Wagenmakers et al., 2007) to the data. Going beyond common behavioral analyses of mean accuracy and response time, the EZ-diffusion model estimates three parameters to decompose the behavioral data into potential underlying processes that might account for individual and age-related differences in working memory performance. Specifically, the drift rate parameter (*v*) reflects the impacts of information quality on the efficiency of evidence accumulation during two-alternative forced choice tasks, whereas the boundary separation (*a*) and non-decision time (*ter*) parameters, respectively, capture response cautiousness and the sensorimotor non-decision processes. The three parameters are estimated from the behavioral data by taking into account both accuracy and response time measures, thereby also handling the issue of speed-accuracy tradeoff. The diffusion model was initially applied to lexical decision tasks that require the subjects to decide between words and non-words, i.e., involving semantic memory processes. Later it has also been applied to tasks requiring short- or long-term memory (cf. Wagenmakers et al., 2007 for review). In the context of the *n*-back task, classifying targets versus non-targets requires the retrieval of items presented in the previous *n* trials from working memory in order to decide whether the item on the current trial is a target or a non-target. Accordingly, depending on the *n*-back load, decision making in the given *n*-back task is informed by memory (Bornstein et al., 2018), which would also be reconcilable with the multi-component model of working memory (Baddeley, 2003; see also Jeneson and Squire, 2012) as well as with more recent evidence on memory-based decisions beyond working memory (cf. Weilbächer and Gluth, 2017 for review). The distinctiveness (quality or memory strength) of the retrieved item representations from previous trials affects the memory evidence accumulation process, which is reflected in the drift rate parameter (*v*).

In terms of hypotheses, we first expected an overall slowing in older age (Salthouse, 1992), which should be reflected in larger non-decision times in older compared to younger adults irrespective of the reward manipulation. Second, in light of dopamine’s role in reward processing (Schultz, 2015) and age-related decline in dopamine modulation (Bäckman et al., 2011; Li and Rieckmann, 2014), we hypothesized that the effects of reward anticipation would be larger in younger compared to older adults. Specifically, we expected age differences in the effects of reward cueing on facilitating selective encoding and retrieval of the relevant target information through incentivized saliency of reward cued items, thus in improving the rate of evidence accumulation or in increasing response cautiousness (prioritizing accuracy over speed) through more salient context representation of opportunity costs (Beeler and Mourra, 2018; errors in trials associated with anticipated rewards are associated with higher cost). Lastly, in a more exploratory manner we also assessed cognitive processing fluctuation, basic memory storage capacity, and subjective reward sensitivity in order to examine potential behavioral correlates of the reward anticipation effects. Of specific interest, previous theoretical (Li et al., 2001, 2006) and empirical evidence (cf. MacDonald et al., 2009 for a review) suggests that intraindividual cognitive processing fluctuations reflect individual and age-related differences in dopamine modulation and related cognitive performance in older adults (MacDonald et al., 2012). We therefore expected cognitive processing fluctuations to be negatively correlated with reward-related performance benefit in the reward incentivized *n*-back task, especially in the high working memory load condition which requires additional cognitive resources for attentional focus switching (McElree, 2001).

## Materials and Methods

### Participants

A total of 50 participants, including 24 younger and 26 older adults (12 females each) were recruited from a population-based database provided by the local registry office of the city of Dresden for research purposes. Before participating in the experimental tasks, all participants were screened for current physical, neurological, and psychiatric health conditions as exclusion criteria. Older participants further underwent dementia screening using the Montreal Cognitive Assessment (MOCA; Nasreddine et al., 2005). In accordance with the recommendation of a recent meta-analysis on the MOCA cutoff score that differentiates healthy aging from possible mild cognitive impairment or dementia (Carson et al., 2018), we applied the inclusion criteria of at least 23 points from the MOCA total score of 30 points (MOCA_mean_ of the final older adult sample = 26.8 points). Altogether, three younger and three older adults were excluded, resulting in a final sample of 21 younger (age_mean_ = 22.7 years, 10 females) and 23 older adults (age_mean_ = 71.0 years, 12 females). The reasons for the study exclusions were (i) incomplete study participation/data (three young and one older adult), (ii) cognitive screening failure (one older adult), and (iii) pre-existing neurological disease (one older adult). All study participants reported normal or corrected-to-normal vision. At the end of the experiment, all study participants received 7.5 Euro per hour as s compensation for study participation and an additional bonus from the reward condition of the working memory task. All subjects gave written informed consent in accordance with the Declaration of Helsinki (2008). The study’s procedures were approved by the ethic committee of the TU Dresden, Germany (EK 276072013).

Demographic characteristics and basic cognitive covariates of the sample are provided in **Table 1**. Gender distributions as well as the total years of education were comparable between the two age groups. The number of years of education did not differ statistically between the two age groups; however, since it is not common for individuals of the older cohort to obtain university (or equivalent level of) education, the data might hint toward a slight positive selection bias of the older adult sample. To further assess the participants’ basic intellectual abilities independently from the main experimental task, we used the computer-based Identical-Pictures (IDP) and Spot-a-Word (SAW) tasks as measures of fluid and crystallized abilities, respectively (Lindenberger and Baltes, 1997). In line with previous findings from a population-based lifespan sample (Li et al., 2004), younger adults showed markedly faster response times [RTs; ΔRT = 1338.3 ms, *t*(32) = -9.5, *p* < 0.0001] as well as higher accuracy [ΔAccuracy = 12.5, *t*(42) = 10.4, *p* < 0.0001] in the IDP task than older adults. In contrast, older adults outperformed younger adults in the SAW task for verbal knowledge [ΔAccuracy = 8.1; *t*(42) = -5.6, *p* < 0.0001]. These results reflect the expected aging-related decline in basic cognitive speed and experience-related increase in semantic verbal knowledge in our sample. Of note, the two age groups also did not differ with respect to the subjective reward responsiveness (*p* = 0.27) as measured by the BIS/BAS questionnaire (Carver and White, 1994; Strobel et al., 2001). Further details about the key experimental tasks and questionnaires are provided in the following section.

**Table 1:** Demographic characteristics and cognitive covariate data by age group.

Variable^∗^	Younger adults (*n* = 21)	Older adults (*n* = 23)	Test statistic	*P*-value
Age range (years)	20–27	65–78		
Mean age (years)	22.7 (1.9)	71.0 (3.6)	*t*(34) = -56.4	**<0.0001**
Gender (females : males)	10 : 11	12 : 11	χ^2^(1) = 0.1	1.00
Education (years)	15.7 (1.2)	16.1 (3.4)	*t*(28) = -0.5	0.60
MOCA	N/A	26.8 (1.8)		
SAW (number correct)	17.6 (4.8)	25.7 (4.7)	*t*(42) = -5.6	**<0.0001**
IDP (number correct)	34.5 (4.2)	22.0 (3.7)	*t*(42) = 10.4	**<0.0001**
IDP (RT correct, ms)	2006.3 (285.3)	3344.6 (605.9)	*t*(32) = -9.5	**<0.0001**
IDP (processing fluctuations^1^, ms)	673.8 (163.6)	1010.3 (265.5)	*t*(42) = -5.0	**<0.0001**
DS-forward (number correct)	8.3 (1.8)	7.5 (1.5)	*t*(40) = 1.6	0.12
DS-backward (number correct)	8.8 (2.2)	7.2 (1.9)	*t*(40) = 2.4	**0.02**
BAS reward responsiveness	3.3 (0.4)	3.2 (0.4)	*t*(42) = 1.1	0.27

*^∗^If not otherwise specified, values indicate means with standard deviations in parenthesis. BAS, Behavioral Approach System (of the BIS/BAS scale); DS, Digit-Span test; IDP, Identical-Pictures task; MOCA, Montreal Cognitive Assessment; SAW, Spot-a-Word task. ^*1*^Processing fluctuation in the IDP task are presented as trial-by-trial standard deviation to illustrate the age group differences. Bolded values indicate significant effects with *p* < 0.05.*

#### Power Calculation

In order to appraise the required sample size, we conducted a power calculation based on effect size estimates of prior empirical work. Given that, to our best knowledge, reward modulation of working memory performance has so far only been investigated in young adults, we based the power calculation on studies reporting main effects of reward on working memory in younger adults (aged 19–37 years) using different paradigms (Krawczyk et al., 2007; Beck et al., 2010; Capa et al., 2013; Klink et al., 2017). First, the effect sizes (partial eta-squared, henceforth referred to as η^2^) were retrieved from the respective publications or, if not provided, estimated from the reported *F*-statistic and the degrees of freedom of the main effects of reward (Maxwell et al., 1981; Lakens, 2013). The effect size estimates ranged from 0.24 to 0.87 (medium to large) in young adults. Using G^∗^Power (version 3.1.9.3), η^2^ effect size estimates were converted to generic effect sizes *(f)* ranging from 0.56 to 2.59. In the next step, we entered the minimum effect size for observing a main effect of reward on working memory performance in younger adult (*f* = 0.56) into an *a priori* power analysis for repeated-measurement between-within ANOVA designs (considering the between-subject factor age group and the within-subject factor reward manipulation). The power analysis was conducted with *α* = 0.05 (two-tailed significance level), 1–β = 0.95 (statistical power), and *r* = 0.5 (correlation between repeated measurements). The results indicated a minimum sample size of 14 participants to sufficiently detect medium effect sizes of reward. However, given that effect sizes are expected to be smaller in older adult samples, we increased the respective sample sizes to at least 20 participants per group, which is sufficient to detect effect sizes of η^2^ > 0.15.

### Main Experimental Task, Study Procedure, and Key Measures

The *n*-back task (Kirchner, 1958; Cohen et al., 1997) requires simultaneous storage and updating of information and shows high loadings on a common factor of working memory that include multiple paradigms for assessing complex memory span, sorting span and memory updating (Schmiedek et al., 2014). For this study, we developed a reward modulated *n*-back task using the Psychophysics Toolbox (Psychtoolbox version 3 ^1^) in Matlab to assess working memory in rewarded versus non-rewarded conditions under low and high memory loads.

#### Age-Adjustment of *n*-Back Load

Although the *n*-back task provides valid information about working memory performance in younger and older adults (Schmiedek et al., 2014), task performance is also largely affected by the interaction between age-related decline in working memory capacity and the *n*-back load. Since age-related decline in working memory capacity (e.g., as reflected in age × load interaction) is a well-established phenomenon (e.g., Nagel et al., 2011; Bennett et al., 2013; Klencklen et al., 2017; see also Karbach and Verhaeghen, 2014 for review), the aim of this study is not to further verify this point, but to investigate age differences in the effects of reward anticipation on working memory that are not confounded with differences in performance accuracy between age groups. Therefore, in order to compare the effects of reward in both age groups at a similar performance level, the memory load (low vs. high) was age-adjusted: younger adults performed the 2- and 3-back conditions, whereas older adults performed the 1- and 2-back conditions. The respective ranges of the memory loads for the two age groups were selected based on prior evidence to avoid floor and ceiling effects. Age-adjusted load manipulation with older adults being tested at a lower range of memory loads than younger adults is a common approach used to compare brain correlates or other moderators of age differences in working memory (e.g., Emery et al., 2008). Adjusting the *n*-back load this way still allowed us to directly compare the performances of young and older adults in the 2-back condition.

#### Reward Manipulation and General Task Structure

**FIGURE 1:**
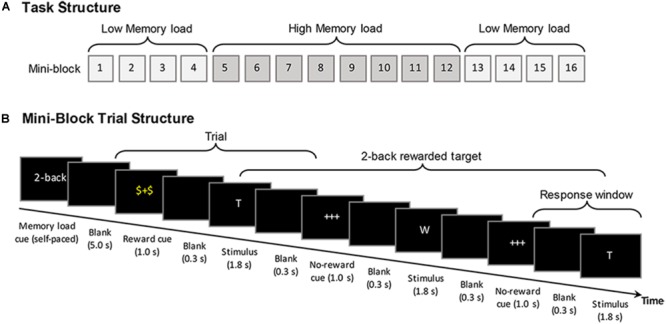
Schematic diagram of the reward modulated *n*-back working memory task. **(A)** The general structure of the task, showing the mini-block sequence that was applied for younger and older adults. **(B)** The trial time course of the task using the example of the 2-back condition (see text for further details).

Altogether, the participants performed 16 blocks in a fixed A-B-B-A order, with 4 mini-blocks of low working memory load followed by 2 times of 4 mini-blocks of high load and again 4 mini-blocks of low load (see **Figure 1A**). The mini-block design was used to reduce potential condition switching costs for older adults (cf. Nagel et al., 2011). Altogether, a total number of 384 stimuli were presented, with 24 stimuli in each mini-block. Each block comprised 30% target stimuli. In each block 50% of the target and non-target stimuli were preceded by the reward cue, whereas the remaining half were preceded by the non-reward cue. The total duration of the task was 23 min. The participants were instructed that, for each of the stimuli following a reward cue they could accumulate 10 bonus points, if they responded correctly n letters later (“yes” responses to targets and “no” responses to non-targets). The size of *n* (i.e., the *n*-back load) was indicated at the beginning of each mini-block. During the brief training before starting with the actual experimental blocks, it was made sure that the subjects understood that the reward did not always refer to their response in the next trial but to their response *n* trials later. A feedback about the accumulated amount of obtained bonus points was presented at the end of each mini-block. While working memory loads (low vs. high) were manipulated at the mini-block level to reduce potential age differences associated with task switching cost, the reward conditions were manipulated at the trial level. The reward manipulation was applied at the trial level within the mini-blocks in order to elicit a stronger involvement of phasic rather than on longer-term tonic reward responses. It has been proposed that short-latency phasic dopamine signals play a crucial role in focused reward-related behavioral adaptations and is relevant for the facilitating effect of incentive salience during learning and decision making (i.e., incentive salience), whereas the tonic dopamine signals are considered to act less specific (cf. Grace, 2000; Schultz, 2002, 2013; Schultz et al., 2017).

At the end of the experiment, participants received 1 Euro Cent per 1 bonus point in addition to the regular compensation for their study participation. It is known that age can affect reward preferences such as for financial versus non-financial rewards (e.g., Sierminska and Takhtamanova, 2007; von Bonsdorff, 2011). Of note, whereas age groups can differ with regard to their motivation for gaining financial rewards, motivation for non-financial rewards remains relatively stable across the adult lifespan (e.g., von Bonsdorff, 2011). We therefore added a non-financial bonus to the rewards *n*-back task, which involved a donation to the German Child Cancer Foundation made by our lab. The amount of donation was equal to the amount of bonus points the subject gained in the rewarded *n*-back task. Since motivation for non-financial bonus is less affected by age, providing this type of reward in addition to the financial bonus could reduce potential age differences in incentive motivation. A donation in the total amount of bonus earned by all participants in the study was given to the foundation after the data collection of the study had been completed.

#### Block-Level Trial Structure

At the beginning of each mini-block, an instruction screen indicated the memory load condition (high or low load) for 5 s. In each mini-block, participants were visually presented with a continuous sequence of capital letters (white font on a black background) selected from a pool of 12 consonants. Before the start of each mini-block, the letter sequences for each mini-block were created pseudorandomly based on probabilistic specifications (30% target stimuli, with 50% of the target and non-target stimuli being rewarded). Therefore, the exact number of trials for each condition varied (i.e., ±1 target) from mini-block to mini-block, with an average of 115.5 target trials across blocks. At the beginning of each trial, a cue was presented for 1000 ms that indicated whether or not the following stimulus would be rewarded (with 10 bonus points), if later correctly responded. A “$+$” cue presented in the color yellow indicated a stimulus to which the correct response would be rewarded, whereas a “+++” cue presented in white indicated a stimulus to which the response would not be rewarded. After the cue, following a 300 ms blank screen the stimulus was presented for a fixed duration of 1800 ms. During the presentation of the stimulus, the participants were required to indicate whether the current stimulus matched the stimulus presented *n* trials ago by pressing either a green button for “yes” (target) or the red button for “no” (non-target) on a response pad with their left or right index finger, respectively (the locations of the response keys were counter-balanced between participants). The participants had time to response until a next blank screen that appeared for 300 ms. Including cue presentation a trial took altogether 3400 ms, after which the cue for another trial followed (see **Figure 1B**).

### Study Procedure

All participants took part in a 1.5- to 2-h laboratory session. After completing the demographic questionnaire, the participants received the instructions for the reward modulated *n*-back task and a brief training to familiarize themselves with the task procedures and the response pad. The cognitive covariates (i.e., digit span, the IDP and the SAW tasks) and a measure of subjective reward sensitivity (Carver and White, 1994) were assessed after the main experimental task was completed.

### Measures of Working Memory Performance in the Reward Modulated *n*-Back Task

Each participants’ accuracy in the *n*-back working memory task was computed by taking the ratio between the sum of the numbers of correct hits and correct rejections divided by the total number of responded trials (i.e., the total number of trials minus the number of trials participates did not respond). Trials were classified as correct hits if participants correctly responded “yes” when the current stimulus matched the stimulus *n* trials ago. Similarly, trials were classified as correct rejections when participants correctly responded “no” in case the current stimulus did not match the stimulus *n* trials ago. Response time (RT) was computed as mean RT, while excluding trials with RTs below 150 ms. Accuracy and RT were computed separately for both memory load conditions (low vs. high) and for the rewarded and non-rewarded trials.

Other than the raw behavioral data, we further modeled the accuracy and RT (mean and variance) data using the EZ-diffusion model for 2-alternative force-choice RT tasks (Wagenmakers et al., 2007) to take potential speed-accuracy tradeoffs into account as well as to decompose performance into subprocesses of the memory-based decision. The EZ-diffusion model is a simplified model of the Ratcliff diffusion model (Ratcliff, 1978); it does not rely on a parameter fitting routine but derives three unobserved process parameters by taking performance accuracy, RT and the variance of RT jointly into the derivations. Specifically, the EZ-diffusion model decomposes the behavioral data into three process parameters: drift rate, (*v*), boundary separation (*a*), and non-decision time (*ter*). In the context of memory research (Ratcliff et al., 2004; Spaniol et al., 2006), these parameters are commonly taken to reflect: (i) the quality of the match between the test stimulus and memory which affects the memory evidence accumulation process for 2-alternative choice decision (*v*), (ii) the stringency (cautiousness) of the decision criterion in choosing the “yes vs. no” response (*a*), and (iii) the non-decision time (*ter*) reflecting sensorimotor processing speed. The model was run with a Matlab-based script. Specifically, we applied the model to each participant’s accuracy and RT data from the rewarded and non-rewarded trials in the high and low memory load conditions to derive the three model parameters for each of the load by reward conditions at the individual level.

### Measure of Basic Memory Storage Capacity

To quantify the participants’ basic memory capacity independently from the *n*-back task, we applied the Digit-Span test (Wechsler Adult Intelligence Scale subtest; Wechsler, 1981). Participants were orally presented with sequences of digits and asked to repeat each sequence either in the exact serial order (forward digit span condition) or in reversed order (backward digit span condition) without delay. The difficulty level was gradually enhanced by increasing the sequence length (3 to 9 digits for the forward condition and 2 to 8 digits for the backward condition). Each difficulty level was presented twice. Both digit span conditions ended after two consecutive errors within one difficulty level. The sum score of correctly performed items was computed for forward and backward digit span, respectively (the maximum score was 14 points in each of the two conditions).

### Measure of Intraindividual Cognitive Processing Fluctuation

Intraindividual fluctuation in cognitive processing time is substantially higher in childhood and old age, relative to young adulthood (Li et al., 2004; Papenberg et al., 2013). It is also predictive of aging-related individual differences in tasks requiring executive control functions (e.g., Lövdén et al., 2007). Furthermore, findings from empirical studies (MacDonald et al., 2009, 2012) and theoretical work (Li et al., 2001, 2006) suggest that intraindividual fluctuation in cognitive processes is a behavioral correlate of the efficacy of dopamine modulation of the fidelity of neuronal information processing. In light for its correlational relevance in reflecting aging-related changes (Lövdén et al., 2007) and brain electrophysiological oscillations in the theta band (Papenberg et al., 2013) during cognitive control, we assessed intraindividual processing fluctuation using the IDP task. The IDP presents simple black-and-white line drawings on a computer screen and requires participants to match the target stimulus to one of the five stimuli presented below the target stimulus by pressing the respective key (1 to 5) as fast and as accurately as possible. The IDP task was limited to a maximum duration of 80 s. This task reflects basic cognitive processing speed and is commonly taken as one of the markers of fluid intelligence (Lindenberger and Baltes, 1997). In the following, we computed intraindividual processing fluctuation as the coefficient of variance [i.e., the standard deviation (SD) of the trial-by-trial response times (RTs) divided by the mean RT].

### Measure of Subjective Reward Sensitivity

Given that we manipulated incentive motivation in the *n*-back working memory task, we further assessed potential age-related differences in subjective reward sensitivity using the behavioral approach system (BAS) scale (Carver and White, 1994). We specifically focused on the reward responsiveness (BAS-RR) subscale of the German version of the BIS/BAS questionnaire (Strobel et al., 2001). The BAS-RR subscale includes statements such as: “When I get something I want, I feel excited and energized,” “When I see an opportunity for something I like I get excited right away”. The participants were asked to indicate to what degree they agreed or disagreed with each statement on a 4-point Likert scale ranging from 1 (very true for me) to 4 (very false for me).

### Statistical Analyses

All analyses were conducted using IBM SPSS software (version 24). Relevant demographic characteristics and covariate measures were compared between the two age groups using Student’s *t*-test for normally distributed continuous variables (or Welch’s *t*-test in case of unequal variances between groups) and Pearson Chi-square (χ*^2^*) test for categorical variables. Age group differences in measures from the *n*-back task [accuracy, mean RT, RT variability, and diffusion model parameters] were examined in separate repeated measurement analysis of variance models (ANOVA) with Age Group (younger vs. older adults) as the between-subject factor and memory Load (low vs. high) as well as Reward Condition (rewarded vs. not rewarded) as within-subject factors. *Post hoc* statistical analyses were conducted with second level ANOVA analyses and pairwise comparisons using the Student’s *t*-tests, where applicable. Correlational analyses were carried out for the two age groups separately using Pearson’s correlation coefficient (*r*), reporting *p*-values both uncorrected and corrected according to Holm (1979). Normal distribution of variables and model residuals were examined using the Shapiro–Wilk test. Effect sizes are reported as partial eta-squared (η^2^). For all statistical tests, the level for statistical significance was set to alpha (α) = 0.05.

## Results

In the following, we first present results from the repeated-measure ANOVA models that address effects of reward modulation (rewarded vs. not rewarded), age (younger vs. older), and memory load (low vs. high load) on working memory performance for accuracy, RT, and process parameters derived from the EZ-diffusion model. Afterward, correlational analyses examining the relations between individual differences in reward-related enhancement of working memory and other behavioral correlates are presented. As age effects on memory load-dependent performance have already been very well-established (see “Introduction” and rationale for the age-adjusted load manipulation in the “Materials and Methods” section), we focus first on the results from analyses of age-adjusted memory load (1- vs. 2-back and 2- vs. 3-back as low and high loads for older and younger adults, respectively). Results regarding RT and accuracy from ANOVA models considering only the 2-back condition, i.e., with both age groups performing the *n*-back task with the same memory load requirements, are reported afterward, followed by the exploratory correlational analyses.

### Effects of Reward Modulation and Age on Accuracy and RT

**FIGURE 2:**
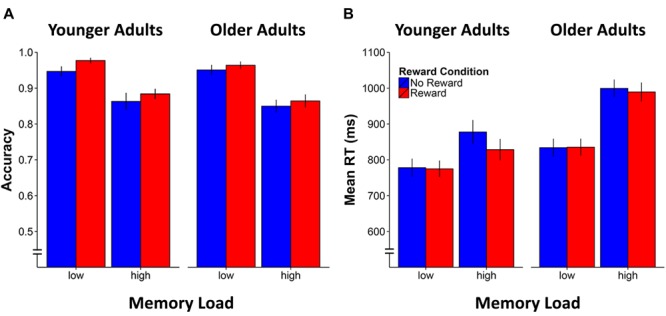
Mean accuracy **(A)** and response time (RT) performance **(B)** for younger and older adults by working memory load and reward condition. Error bars represent standard errors of the mean.

For accuracy, the analysis showed that the age-adjusted load manipulation was effective, resulting in both age groups performing at a similar level (*p* = 0.6 for main effect of age). The analysis also revealed the expected significant main effect for Memory Load [*F*(1,42) = 121.8, *p* < 0.0001, η^2^ = 0.7]. Of particular interest, the main effect of Reward Condition [*F*(1,42) = 10.0, *p* = 0.003, η^2^ = 0.2] was also significant, indicating enhanced *n*-back performance accuracy for the rewarded compared to non-rewarded stimuli, irrespective of age group or memory load (see **Figure 2A**). The two-way interactions involving Memory Load × Reward Condition and Age Group × Reward Condition as well as the three-way interaction were all not significant (*p*s > 0.3).

As for effects on RT, an equivalent repeated-measure ANOVA showed significant main effects of Age Group [*F*(1,42) = 8.9, *p* = 0.005, η^2^ = 0.2], Memory Load [*F*(1,42) = 80.2, *p* < 0.0001, η^2^ = 0.7], and Reward Condition [*F*(1,42) = 10.0, *p* = 0.03, η^2^ = 0.2]. Similarly, the two-way interactions of Age Group × Memory Load [*F*(1,42) = 9.9, *p* = 0.003, η^2^ = 0.2], Age Group × Reward Condition [*F*(1,42) = 5.1, *p* = 0.03, η^2^ = 0.1], and Memory Load × Reward Condition [*F*(1,42) = 15.4, *p* < 0.0001, η^2^ = 0.3] as well as the three-way interaction of Age Group × Memory Load × Reward Condition [*F*(1,42) = 5.5, *p* = 0.02, η^2^ = 0.1] were all significant. These results indicate that reward facilitated response speed (reduced RT) only in the high memory load condition (see **Figure 2B**), and specifically in the younger [*t*(20) = -3.8, *p* = 0.001] but not in older adults [*t*(22) = -1.4, *p* = 0.2]. Further analysis of the RT variability during the *n*-back task (i.e., the standard deviation of RT during correct responses) revealed a main effect of Memory Load indicating higher variability in the high compared to the low *n*-back load condition [*F*(1,42) = 42.6, *p* < 0.0001, η^2^ = 0.5], as well as an Age Group × Memory Load interaction [*F*(1,42) = 4.6, *p* = 0.04, η^2^ = 0.1]. Accordingly, the increase of RT fluctuations in the high compared to the low *n*-back load condition was more pronounce in older compared to younger adults irrespective of the reward manipulation [*t*(42) = 2.1, *p* = 0.04]. No effect of reward manipulation could be observed. Repeating the ANOVA model but considering the RT variability across all responses (correct and erroneous) did not reveal any additional results.

### Effects of Reward Modulation and Age on Parameters of Diffusion Model

As the diffusion model takes both accuracy and RT into account and decomposes performance into subprocesses, we conducted three separate repeated measurement ANOVAs to investigate the effects of age and reward manipulation on the three model parameters drift rate (*v*), boundary separation (*a*), and non-decision time (*ter*). The within- and between-subject factors were identical as in the analyses of the raw behavioral data.

**FIGURE 3:**
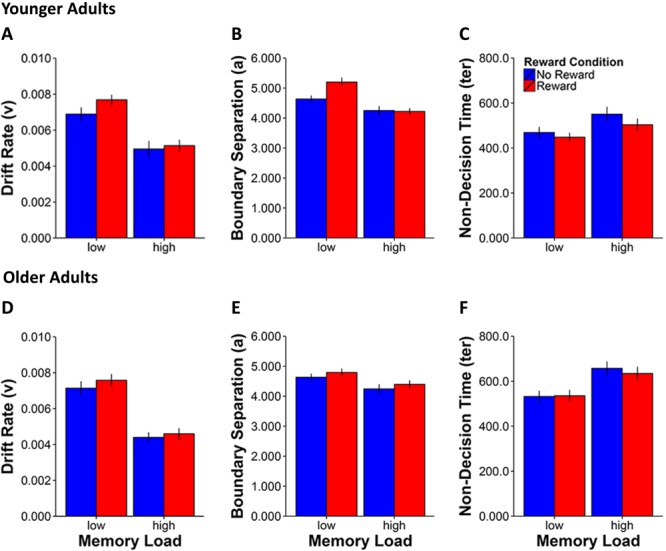
Mean values for the three EZ-diffusion model parameters drift rate **(A,D)**, boundary separation **(B,E)**, and non-decision time **(C,F)** for younger **(A–C)** and older adults **(D–F)** by working memory load and reward condition. Error bars represent standard errors of the mean.

For the drift rate parameter (see **Figures 3A,D**), we observed main effects for Memory Load [*F*(1,42) = 151.9, *p* < 0.0001, η^2^ = 0.8] and Reward Condition [*F*(1,42) = 8.2, *p* = 0.007, η^2^ = 0.2] as well as a Memory Load × Reward Condition interaction [*F*(1,42) = 5.0, *p* = 0.03, η^2^ = 0.1], indicating a greater reward anticipation related improvement in the rate of memory evidence accumulation in the low load condition in both younger [*t*(20) = 2.3, *p* = 0.03] and older adults [*t*(22) = 2.3, *p* = 0.03].

Regarding the boundary separation parameter (see **Figures 3B,E**), significant effects were found for Memory Load [*F*(1,42) = 38.5, *p* < 0.0001, η^2^ = 0.5], Reward Condition [*F*(1,42) = 15.2, *p* < 0.0001, η^2^ = 0.3] and a two-way Memory Load × Reward Condition interaction [*F*(1,42) = 9.2, *p* = 0.004, η^2^ = 0.2]. Furthermore, the three-way Age Group × Memory Load × Reward Condition interaction was also significant [*F*(1,42) = 8.8, *p* = 0.005, η^2^ = 0.2]. Together these results indicate increased response cautiousness for rewarded compared to non-rewarded stimuli in the low Memory Load condition in younger [*t*(20) = 4.0, *p* = 0.001] but not in older adults [*t*(22) = 1.6, *p* = 0.1].

Analysis of the last parameter which characterized non-decision time (*ter*) that mostly reflects sensorimotor speed (see **Figures 3C,F**) showed again main effects of Memory Load [*F*(1,42) = 58.9, *p* < 0.0001, η^2^ = 0.6] and Reward Condition [*F*(1,42) = 11.2, *p* = 0.02, η^2^ = 0.2]. Furthermore, we also found the expected age group effect [*F*(1,42) = 8.5, *p* = 0.006, η^2^ = 0.2], which reflects the overall sensorimotor slowing of older compared to younger adults irrespective of manipulations of memory load and reward modulation. The two-way interaction of Memory Load × Reward Condition [*F*(1,42) = 6.3, *p* = 0.02, η^2^ = 0.1] was also significant, indicating a greater effect of reward incentive in reducing non-decision times in the high than in the low memory load condition both in younger [*t*(20) = -2.9, *p* = 0.009] and in older adults [*t*(22) = -2.2, *p* = 0.04].

### Results Regarding Effects of Age and Reward Manipulation in the 2-Back Condition

We further analyzed effects of age and reward effects in reduced ANOVA models for accuracy, RT, and diffusion model parameters solely in the 2-back condition (i.e., the low-load condition for younger adults and the high load condition for the older adults).

As would be expected in an aging sample for both accuracy and RT, a clear age effect was found indicating a reduced number of correct responses [*F*(1,42) = 29.3, *p* < 0.0001, η^2^ = 0.4] and increased reaction times [*F*(1,42) = 41.5, *p* < 0.0001, η^2^ = 0.5] in older compared to younger adults. Whereas accuracy was increased in the 2-back condition for rewarded stimuli irrespective of age group [*F*(1,42) = 11.1, *p* = 0.02, η^2^ = 0.2], no main effect of Reward Condition could be observed for response time. The Age Group × Reward Condition was also not significant either for accuracy or RT (*p*s > 0.2), when only considering the 2-back condition.

Similar to the accuracy and response time data, the reduced models revealed clear age effects for the drift rate and the non-decision time parameter. Given the observed main effects, older adults showed more deficits in memory evidence accumulation [*F*(1,42) = 56.5, *p* < 0.0001, η^2^ = 0.6] and increased non-decision times [*F*(1,42) = 28.8, *p* < 0.0001, η^2^ = 0.4] compared to younger adults. Also, reward facilitated memory evidence accumulation [*F*(1,42) = 7.5, *p* = 0.009, η^2^ = 0.2] and reduced non-decision time [*F*(1,42) = 7.6, *p* = 0.009, η^2^ = 0.2] irrespective of age group. In line with the results of the full models, only for the boundary separation parameter we observed a significant Age Group × Reward Condition interaction [*F*(1,42) = 6.5, *p* = 0.01, η^2^ = 0.1], hinting toward a reward-related increase in response cautiousness in younger [*t*(20) = 4.0, *p* = 0.001] but not in older adults [*t*(22) = 1.8, *p* = 0.09].

### Correlational Analysis of the Associations Between Reward-Induced Benefits in RT and Covariates

**FIGURE 4:**
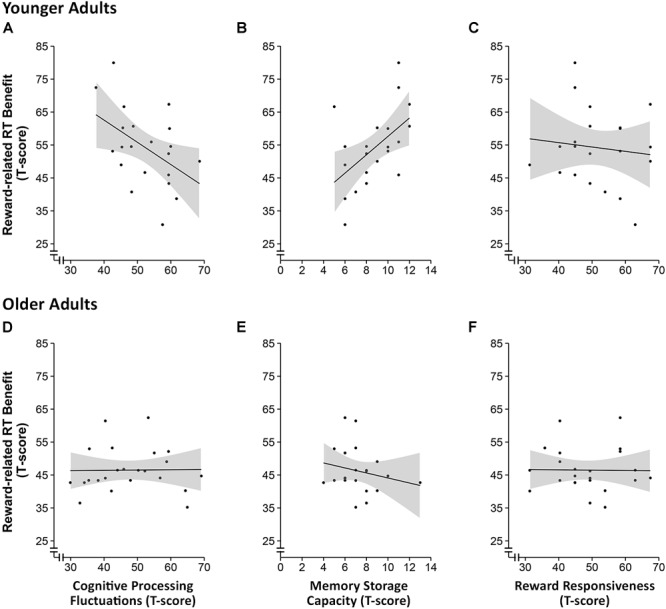
Scatter plots of the correlational analysis of the reward-related response time benefit and intraindividual fluctuations in cognitive processing **(A,D)**, working memory storage capacity **(B,E)** and reward responsiveness **(C,F)** in younger **(A–C)** and older adults **(D–F)**. The shaded areas around the curves represent the 95% confidence interval.

Based on the behavioral data from the *n*-back task showing that the effect of reward cue on RT was stronger in the high load condition, we computed a reward-induced gain score as the difference between the mean RT for non-rewarded trials and the mean RT for rewarded trials in the high memory load condition. Pearson correlation coefficients were then computed to investigate potential associations between individual differences in RT reduction due to reward incentive and individual differences in basic cognitive processing fluctuation, memory storage capacity (focusing on the more effortful backward digit span), and reward sensitivity separately for the two age groups. We found that reward incentive induced RT benefits in younger adults were negatively correlated with cognitive processing fluctuations (*r* = -0.5, *p* = 0.03, *n* = 22), which remained marginally significant after correcting for multiple correlations according to Holm (1979; *p*_corrected_ = 0.06). Reward incentive induced RT benefits in younger adults were further positively correlated with memory storage capacity in the harder, backward digit span condition (*r* = 0.5, *p* = 0.01, *n* = 20; *p*_corrected_ = 0.04). No such associations could be observed in the older adult sample (*p*s > 0.3; see **Figure 4**). Furthermore, no association between reward-related RT gain and subjective reward sensitivity (BAS-RR) was observed either.

Since a three-way interaction (Age Group × Memory Load × Reward Condition) was also found with respect to the boundary separation (*a*) parameter of the diffusion model, which considers both RT and accuracy measures, we computed an equivalent gain score for the *a* parameter reflecting the reward-related increase in response cautiousness in the low memory load condition. However, the boundary separation parameter showed no associations with basic cognitive processing fluctuation, memory storage capacity, or reward sensitivity in either age groups.

## Discussion

This study compared the effects of reward modulation on working memory in younger and older adults. As intended by applying an age-adjusted working memory load manipulation, performance accuracy was comparable between both age groups, whereas response times reflected a general age-related slowing (Salthouse, 1992) as expected. In the further analyses, effects of reward incentive on performance accuracy, response time, and the parameters derived from the EZ-diffusion model could be compared between the two age groups at the same accuracy level for the low versus the high working memory load conditions to avoid age differences in baseline performance as a confound (cf. Emery et al., 2008).

Results based on raw behavioral data showed that working memory accuracy in trials that required a decision (target vs. non-target) for a stimulus that was preceded by the reward cue *n* trials before was higher than in trials without the preceding reward cue irrespective of working memory and age group. The aspect in which older adults benefited less than younger adults through reward incentive was response speed. The three-way interaction involving reward, working memory load, and age group showed that reward incentive speeded up younger adults’ working memory-based choices (target vs. non-target) particularly when working memory load was high, but this was not the case for older adults.

However, results from analyses based on parameters derived from the EZ-diffusion model (Ratcliff, 1978; Wagenmakers et al., 2007), which jointly takes into account the individuals’ accuracy as well as the mean and variance of the RT distribution, revealed a more refined pattern of effects of reward incentive on working memory and the associated age differences. The effects of reward anticipation following reward context cueing interacted with working memory (*n*-back) load, such that the effects of the reward cue affected the parameters reflecting cognitive processes of working memory-based decision making – i.e., rate of evidence accumulation (*v*) and cautiousness of response (*a*) – more in the low load condition but the non-decision time parameter (*ter*), which captures sensorimotor processing speed, more in the high load condition. This seems to suggest that the benefit of incentive motivation on selective filtering and updating of information in working memory saturates when the load of working memory maintenance is high, whereas its effect on sensorimotor speed does not dependent on working memory load. Of note, the effect of the reward cue in increasing drift rate (*v*) and in reducing non-decision time (*ter*) was comparable in younger and in older adults, whereas the reward incentive increased the decision criterion (*a*) resulting in more cautious responses only in the younger adults but not in the older adults.

Together these findings indicate that the pre-stimulus incentive cue may selectively prioritize attention resource for encoding the item following the cue and may prime the updating of task context representation in working memory. This interpretation is in line with previous evidence highlighting the relevance of reward context (Kobayashi and Schultz, 2014) in facilitating encoding of target vs. non-target information and the role of striatal dopamine in updating working memory context representations in the prefrontal cortex (D’Ardenne et al., 2012). Furthermore, the current result of age-independent reward anticipation benefits (i.e., the benefit from reward context cueing) on working memory accuracy and the quality (or distinctiveness) of information in working memory (as reflected in the parameter of evidence accumulation rate) suggests that reward facilitating effect on selective attention by raising the saliency of content representations in working memory is still preserved in the young-old age range (65 to 78 years of age in the current sample). In contrast, unlike younger adults, the older adults did not become more cautious in their responses in the rewarded context with higher opportunity costs (Beeler and Mourra, 2018; errors are associated with more costs in trials cued with rewards). We think this effect might reflect that reward facilitation of updating context representations in working memory is impaired in old age.

Results of the correlational analyses show that in younger adults, the benefit of reward anticipation on the RT of making working memory-based decisions correlated with individual differences in cognitive processing fluctuations (measured by the Identical-Pictures task) and working memory storage capacity (measured by the digit span task), but not with a subjective measure of reward sensitivity (measured by the BAS-RR scale). Younger adults with lower processing fluctuations and larger memory storage capacity benefited more from the reward incentive when working memory load was high. However, such relations were not observed in older adults, which could in part reflect the fact that the speed enhancing effect of reward anticipation is rather limited in older adults.

### Limitations

The following limitations should be considered for our study. First, the older participants in our sample might be more positively selected than the younger participants. Albeit both age groups had a similar education level, our results could underestimate some of the age effects. Second, although the reward manipulation was carried out using both financial and non-financial bonuses (i.e., monetary reward and a donation to a non-profit organization in the amount of bonus points gained in the rewarded *n*-back trials) to minimized potential confounding effects of reward type on age differences in reward-induced effects, the two age groups might still have differed in the perceived value of the overall reward. The impact of such difference might be small, since we did not observe age differences in reward responsiveness as indicated by the BAS-RR scale (*p* = 0.3; see **Table 1**). However, given that we did not explicitly ask the subjects to rate both reward types, our finding cannot speak directly to the potential age differences in the motivation for these two types of rewards. Third, although based on hierarchical latent factor modeling the *n*-back task provides a valid assessment of working memory (cf. Schmiedek et al., 2014), individual and age group differences in working memory performance are also dependent on the task paradigm and the stimulus material itself. We decided to focus on the letter *n*-back task, which is also rather easy to instruct for different age groups, to implement a continuous reward manipulation. However, our current findings cannot be generalized to working memory concepts beyond the *n*-back paradigm without further studies replicating our results using different working memory tasks or task batteries. Fourth, according to theories of multiple embedded components of working memory (Oberauer and Hein, 2012; cf. Cowan, 2001), the broad and narrow range of attention focus in working memory may invoke different processes. According to this theoretical framework, in the broad attention range (also called the *region of direct access)*, a small limited number of items is maintained for processing, whereas in the narrow range (also called the *focus of attention*) only one item is available for direct manipulation in the ongoing task. This framework further assumes that target information can be immediately retrieved from the attention focus as long as the *n*-back load is smaller or equal to one (also referred to as attention focus process of working memory). However, with increasing *n*-back load the focus must be switched to select and move one item from the short-term storage back into the focus of attention (also referred to as focus switching process of working memory). Given the higher requirements for cognitive resources, the focus switching process is usually associated with lower accuracy and longer response times. Furthermore, when the *n*-back load is larger than one, subjects might also be more susceptible to item familiarity effects as it is often observed in aging samples (McElree, 2001; Schmiedek et al., 2009). In our study, the age-adjustment of accuracy levels for low *n*-back loads (i.e., 1-back of older adults and 2-back for younger adults) and high *n*-back loads (i.e., 2-back of older adults and 3-back for younger adults) limits the interpretation of the observed age group differences since storage and processing demands are not comparable across the 1-, 2-, and 3-back loads: Whereas the underlying mechanisms are assumed to be comparable for the 2-back and the 3-back condition, which constitute the high working memory load, target availability and accessibility are easier and faster for the 1-back compared to the 2-back condition representing the low working memory loads of our paradigm (McElree, 2001). Accordingly, age groups differences in the boundary separation parameter (*v*) of the EZ-diffusion model in the low working memory load condition should be interpreted with caution. Finally, we are aware that the sample sizes of both age groups are rather small for correlational analysis and that further studies with larger samples and covering broader age ranges of adult development are needed to elucidate potential effects of reward modulation on working memory performance and underlying processes. So far, our correlational results only tentatively hint that smaller cognitive processing fluctuations might be related with higher reward-related benefits in *n*-back response time in younger but not in older age when *n*-back load is high.

## Conclusion and Future Directions

Keeping the above limitations in mind, the results of this study extends previous research on aging and reward modulation of episodic memory and attention allocation in working memory. The here reported findings indicate that although reward modulation of working memory is preserved in healthy older adults with a high education level in the young-old age range, not all aspects of working memory as reflected by the EZ-diffusion parameters benefited from reward incentive. Whereas reward anticipation facilitated older adults’ evidence accumulation and sensorimotor processing speed, their responses did not become more cautious with incentive motivation as observed in younger adults. These results may reflect that in old age the mechanism for reward facilitation of content representations is relatively more preserved than the mechanism for reward facilitation of the updating of context representations in working memory. Neurocognitive correlates of these age effects need to be explored in future research in order to shed further lights on how to combine schemes of motivational incentives with cognitive interventions to enhance the plasticity of working memory in old age.

## Data Availability

The raw data supporting the conclusions of this manuscript will be made available by the authors to any qualified researcher.

## Author Contributions

S-CL formulated the research questions, designed the study, discussed the results, and prepared the manuscript. FT designed the study, analyzed the data, discussed the results, and prepared the manuscript. NZ prepared the main task, acquired and analyzed the data, discussed the results, and prepared the manuscript.

## Conflict of Interest Statement

The authors declare that the research was conducted in the absence of any commercial or financial relationships that could be construed as a potential conflict of interest.

## Footnotes

1 www.psychtoolbox.org
